# CNN-Based Brain Tumor Detection Model Using Local Binary Pattern and Multilayered SVM Classifier

**DOI:** 10.1155/2022/9015778

**Published:** 2022-06-27

**Authors:** Morarjee Kolla, Rupesh Kumar Mishra, S Zahoor ul Huq, Y. Vijayalata, M Venu Gopalachari, KazyNoor-e-Alam Siddiquee

**Affiliations:** ^1^Department of Computer Science and Engineering, Chaitanya Bharthi Institute of Technology, Hyderabad, Telangana, India; ^2^Department of Computer Science and Engineering, G. Pulla Reddy Engineering College, Kurnool, Andhra Pradesh, India; ^3^Department of Computer Science and Engineering, Gokaraju Rangaraju Institute of Engineering and Technology, Hyderabad, Telangana, India; ^4^Department of Information Technology, Chaitanya Bharthi Institute of Technology, Hyderabad, Telangana, India; ^5^Department of Computer Science and Engineering, University of Science & Technology, Chattogram, Bangladesh

## Abstract

In this paper, an autonomous brain tumor segmentation and detection model is developed utilizing a convolutional neural network technique that included a local binary pattern and a multilayered support vector machine. The detection and classification of brain tumors are a key feature in order to aid physicians; an intelligent system must be designed with less manual work and more automated operations in mind. The collected images are then processed using image filtering techniques, followed by image intensity normalization, before proceeding to the patch extraction stage, which results in patch extracted images. During feature extraction, the RGB image is converted to a binary image by grayscale conversion via the colormap process, and this process is then completed by the local binary pattern (LBP). To extract feature information, a convolutional network can be utilized, while to detect objects, a multilayered support vector machine (ML-SVM) can be employed. CNN is a popular deep learning algorithm that is utilized in a wide variety of engineering applications. Finally, the classification approach used in this work aids in determining the presence or absence of a brain tumor. To conduct the comparison, the entire work is tested against existing procedures and the proposed approach using critical metrics such as dice similarity coefficient (DSC), Jaccard similarity index (JSI), sensitivity (SE), accuracy (ACC), specificity (SP), and precision (PR).

## 1. Introduction

Brain tumors develop as a result of unregulated and fast cell proliferation. It can be fatal if not addressed in the early stages. Machine learning techniques are used to assist clinicians in detecting brain tumors and making judgments. The progression in the deep learning procedures involving the best classifiers impacted a significant advance in medical image processing in recent years. A brain tumor develops when brain tissues develop abnormally. The malignant tissues outgrow the healthy cells, resulting in a mass of cells that eventually transform into tumors [1]. Magnetic resonance imaging (MRI) has been the gold standard for noninvasive brain tumor identification in the last few decades due to its improved soft tissue contrast [2, 3]. MRIs have a considerable impact on medical image processing and analysis due to their ability to provide high-resolution information about brain structure and abnormalities [4–6]. A malignant brain tumor grows significantly more quickly than a benign tumor and is more prone to spread to other parts of the brain. Primary malignant brain tumors have poor prognoses and greatly affect cognitive abilities as well as quality of life [7]. The analysis of medical images is critical in assisting people in diagnosing various disorders. The advanced medical imaging modalities are commonly used methods for analyzing anomalies in brain tissues, which can aid in the detection of tumors in their early stages [8]. The data are initially extracted from dataset, which contains MR pictures of the brain. The first stage of the work receives image data from various sources and forwards it to the next layer for preprocessing using linear filters. Further normalization and patch extraction are important procedures performed in the preprocessing layer to prepare the image for use by CNN. Convolution is considered as a mathematical and engineering tool which is involved in the next phase of CNN where the process feature extraction is conducted on the input image combined with multilayered support vector machine (ML-SVM) to provide optimal outcomes in the work.

## 2. Related Works

Numerous medical imaging techniques are utilized to obtain information regarding tumors (tumor type, shape, size, location, and so on) that is required for diagnosis [9]. The Fuzzy C-Mean (FCM) method was developed by the authors of [10, 11]. As a means of avoiding the difficulty of determining the number of clusters in an FCM, this technique was devised to pick the pixel intensities and cluster them into two groups. System sensitivity, specificity, and accuracy are all measured to determine how well it works. SVM and FCM techniques were used to generate a hybrid approach for brain MRI categorization by the researchers in [12]. In the early stages of the technique, MRI scan quality is improved using image enhancement technologies such as contrast augmentation and midrange stretch. There are 71 features constructed using the intensity profile, the obtained co-occurrence matrix with new values, and also the Gabor functions after the tumor contour is obtained using a semiautomatic approach in [13, 14]. Because of limitations such as parameter selection or the need for prior knowledge of the images as well as significant calculation times, skull stripping is a common preparation stage in classical discriminative approaches [15]. Deep learning was utilized to construct a classification method for multigrade brain tumors, according to the authors of [16, 17]. The CNN model is used to segment the tumor in this strategy, although the results are limited in accuracy and sensitivity because of the limitations of the CNN model [18]. According to the findings of another study [19, 20], brain tumors can be detected using both hand-created and deep learner features.

The authors of [21–23] did a comparison of naive Bayes, J48 decision tree, and neural network; the downside of this strategy is that it is a conventional approach. The use of machine learning to categorize brain tumors, on the other hand, was proposed by [24, 25]. KNN and SVM were used in this comparison, and the model was found to be 0.95 percent accurate. Because of the automated intelligent system outcomes, the accuracy of this model is higher, but the sensitivity is lower. By integrating support vector machine (SVM) and artificial neural network (ANN), the authors [26, 27] proposed a technique for better precise identification and division of mental tumors; however, despite the high accuracy, the precision was not completely satisfactory [28, 29].

## 3. Methodology and Algorithm

**Figure 1:**
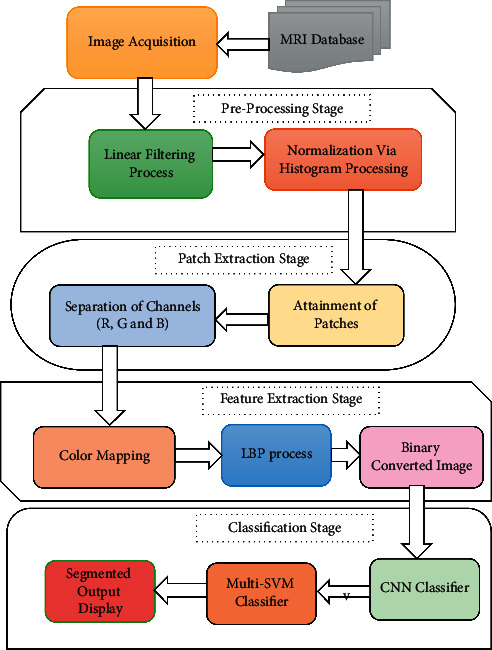
Block diagram of the proposed method.

This study describes a convolutional neural network (CNN) integrated with a multilayered support vector machine (ML-SVM) algorithm. In this system, there are mainly five distinct blocks: Image Acquisition, Preprocessing, Patch Extraction, Feature Extraction, and CNN Classification and ML-SVM Classifier, as shown in Figure 1. Here, the procedure is explained along with each block and also the successive results.

In this process, images are imported from a dataset. This imported image is MRI scan, which is to be from the glioma region of the brain, and this acquired image is processed to detect tumors. The brain scan images are obtained from the Kaggle medical image database, which is the most preferred and standard database in the field of research. The acquired images are then processed by image filtering techniques followed by image intensity normalization as provided in the proposed block diagram. The input images acquired by the medical imaging modalities consist of artifacts due to inherent properties of modalities. These images must be processed at first hand to remove disturbances that have been added and also normalized. Here, the image is subjected to linear filtering action which is accomplished through a neighborhood operation via weight adjustment procedures.

This filtered image is normalized for uniform intensity. Image normalization is carried out by means of contrast adjustment of the pixel values via histogram processing.

The filtered image is then subjected to the patch extraction stage as shown in the proposed block diagram, to provide patch extracted images, where the image is subdivided to obtain separation between channels; in this process, the image is followed through patches attainment which again subdivides each separated channel based on RGB values for red, green, and blue channels, respectively. In feature extraction, the RGB image is converted to a binary image through grayscale conversion via the colormap process, and later, this process is done by local binary pattern (LBP).

A convolutional network can be used to extract feature information, while a multilayered support vector machine (SVM) can be used to recognize objects. CNN is one of the most extensively used deep learning processes in a wide range of engineering applications. As compared to typical feedforward neural networks, CNN contains fewer parameters and connections, which makes training more straightforward. In order to give superior outcomes, this includes the training and testing of the required dataset as the primary process. Because it is an iterative method, it is only stopped when the optimal results are achieved. This model was discovered to be capable of extracting characteristics from raw pictures as well as performing classification tasks on its own. The initial phase, as shown in the proposed block diagram, is to train CNN and multilayered SVM models. The second stage is to run the models through their paces and yield the final segmentation results (Algorithm 1).

## 4. Experimental Investigations

**Figure 2:**
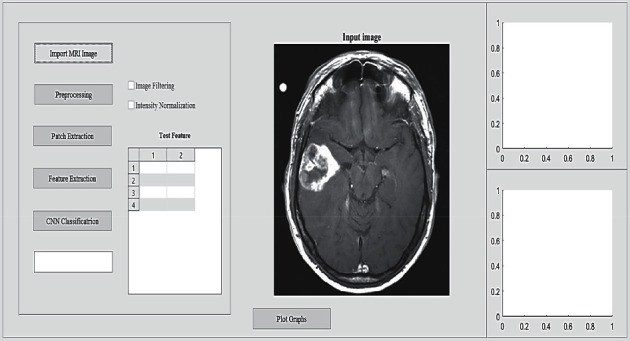
Interface of brain tumor segmentation displaying the stages involved.

The graphical user interface (GUI) to perform the proposed method in order to segment and detect brain tumor with necessary stages involved is shown in Figure 2.

It displays the stages such as image acquisition to import input images from dataset, preprocessing stage to filter the images to remove any unwanted artifacts, patch extraction stage to achieve the patches with respect to channels of RGB, feature extraction stage to carryout color mapping and LBP process to attain the binary image, and CNN classification stage which involves multilayered SVM and later CNN procedures to predict the final output.

**Figure 3:**
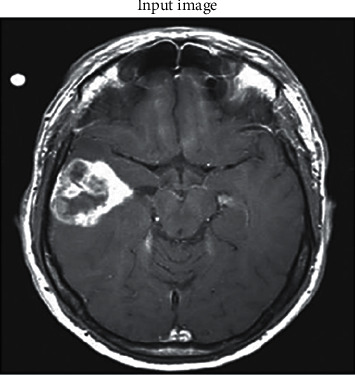
Imported input MRI image from the dataset.

The imported input image is MRI scan, as shown in Figure 3, which is to be from the glioma region of the brain, and this acquired image is processed to detect tumors.

**Figure 4:**
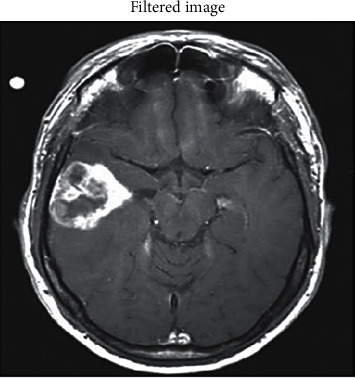
Filtered input MRI image.

The imported image is further fed to the filtering process to attain the filtered output image, as shown in Figure 4. A two-stage filtering procedure is used in this case: first, linear filtering is used, in which the Gaussian filter kernel is employed since it has all of the characteristics of other filters due to the structural organization of its density function; and then, nonlinear filtering is used. It returns a rotationally symmetric Gaussian lowpass filter with a 2D Gaussian smoothing kernel and a positive standard deviation value, resulting in a reduction of artefacts as a result of this filtering.

**Figure 5:**
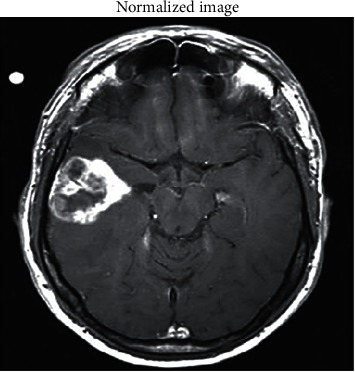
Histogram normalized input MRI image.

The resulting image is then subjected to a normalizing process via histogram processing. An operation called mapping is carried out in this scenario to map the intensity values in the resulting grayscale image to new values. The outcome is that normalizing saturates the lowest and highest one percent of all possible pixel values by increasing the contrast values in the resulting image, resulting in the normalized image displayed in Figure 5.

**Figure 6:**
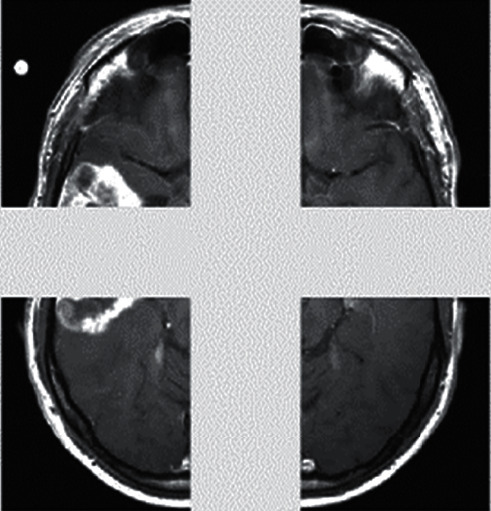
Patch extracted images.

The normalized image is fed into the patch extraction phase, which yields the patch extracted image depicted in Figure 6. Obtaining the patches while keeping the size of each patch in mind is necessary in order to preserve equilibrium. An image patch is a collection of pixels in a photograph that, as the name implies, is a collection of pixels. Patches are divided into groups based on their energy level, with those with a high degree of energy being retained through the use of thresholds.

**Figure 7:**
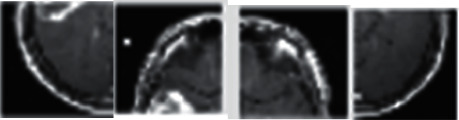
Red channelized images.

**Figure 8:**
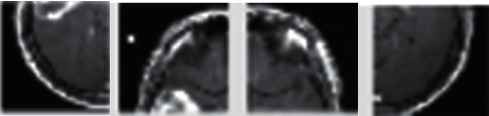
Green channelized images.

**Figure 9:**
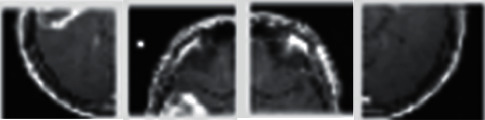
Blue channelized images.

These patches are divided into red, green, and blue channelized images, as shown in Figures 7–9. In contrast to grayscale images, RGB images include three channels. Each pixel is composed of three channels, each representing a different color. It is necessary to analyze the components of each image's primary colors using the RGB channel separation (which is composed of the three colors: red, green, and blue). A mathematical analysis of the image will be performed, and the results will be presented in gray levels for each color, ranging from black to white and from no color to pure color.

**Figure 10:**
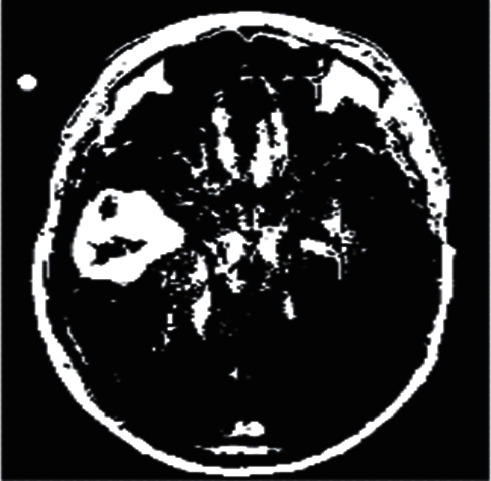
Binary image form of input MRI image.

This channel split image is then subjected to the gray thresholding technique, which performs a basic conversion operation. The LBP process is then launched on the resulting image, with LBP being a form of visual descriptor that is utilized for classification purposes in this context. This simple yet efficient texturing operator labels pixels in an image by thresholding the pixels in their immediate vicinity and treating the result as a binary integer. Local binary pattern (LBP) is one of the most often used texturing operators because of its simplicity and effectiveness. At this point, the output image is a binary transformed image, as illustrated in Figure 10.

**Figure 11:**
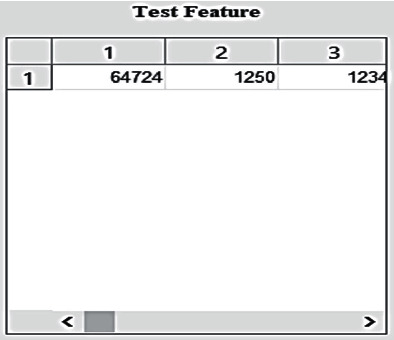
Obtained test features.

Figure 11 depicts the appearance of the obtained test features on the constructed GUI. They are crucial and will be utilized in multilayered SVM and CNN classifiers, among other applications. Supervised machine learning (SVM) is a machine learning technique that can be used to aid in the classification or regression of issues. In order to find the optimal potential boundary between the various outputs, this algorithm is used. Using SVM's most basic version, linear separation, the goal is to find a line that optimizes the separation between two classes of 2-dimensional space points in a two-class dataset.

In its most simple type, SVM does not support multilayered classification natively. Multilayered SVMs are usually implemented by combining several two-class SVMs. Therefore, it is a natural step to go from the standard single-layer SVM to the multilayer SVM.

**Figure 12:**
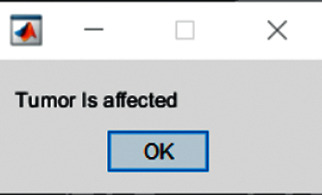
Final output from console.

Later, the classified findings will be appropriately labelled using convolutional neural network (CNN) classifiers. CNN is a deep neural network that is commonly used in image classification and machine vision scenarios. Convolutional neural networks (CNN) are complicated feed forward neural networks used in machine learning. Because of its great accuracy, CNNs are employed for image categorization and recognition. Finally, the console shows a simple message indicating whether or not a tumor is present.  Figure 12 depicts the console output when a tumor is found.

**Figure 13:**
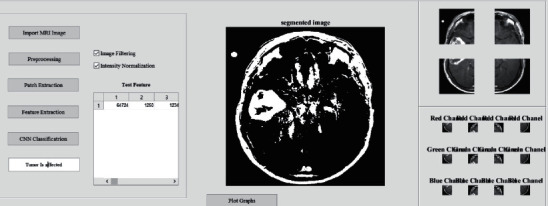
Interface of brain tumor segmentation displaying the stages involved with final outputs for a tumor affected stage.

**Figure 14:**
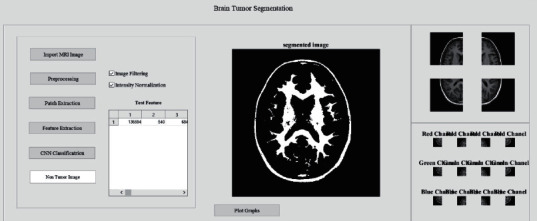
Interface of brain tumor segmentation displaying the stages involved with final outputs for a nontumor stage.

A final result display of the entire work interface of brain tumor segmentation displaying the stages involved with final outputs for a tumor affected stage and also for a nontumor stage is provided in Figures 13 and 14, respectively.

**Figure 15:**
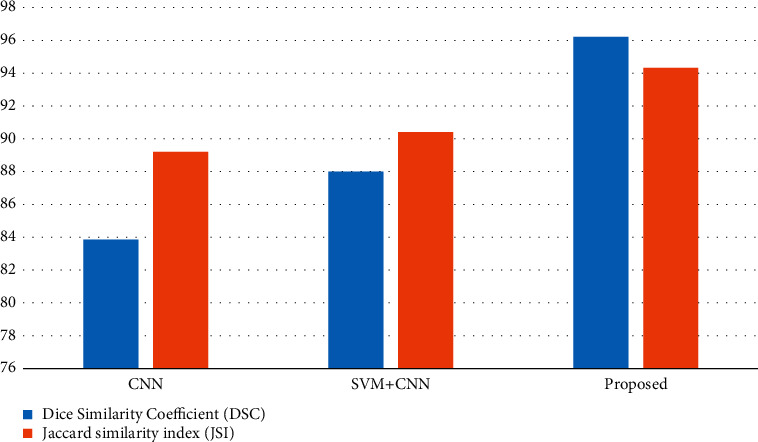
Graphical representation of comparison of DSC and JSI.

The entire work is evaluated for existing procedures as well as the proposed process, and the parameters of importance such as dice similarity coefficient (DSC) and Jaccard similarity index (JSI) are shown in Table 1. The graphical representation of the DSC and JSI comparison is also provided in Figure 15.

In this case, the parameter dice similarity coefficient (DSC) is used to determine the exact amount of ratio of the available real tumor and available nontumor pixels to the anticipated tumor and nontumor pixels and is computed using equation (1), and the Jaccard similarity index (JSI) is used to calculate the percentage of the similarity amid actual tumor pixels in the region of interest and the number of anticipated tumor pixels and is computed as per the standard equation (2).

When the model accurately predicts the positive class, the outcome is known as a “true positive” (TP) in these equations. To denote a result that the model predicted to be positive, the acronym FP stands for false positive, while TN stands for true negative, signifying a result that the model predicted to be negative. When the model inaccurately predicts the negative class, the term “false negative” is used.

(1) $$\begin{array}{c} \text{Dice similarity coefficient }\left(DSC\right)=\frac{\left(2\text{TP}\right)}{\left(\text{FP}+\text{2TP}+\text{FN}\right)}X100, \end{array}$$

(2) $$\begin{array}{c} \text{Jaccard similarity index }\left(JSI\right)=\frac{\left(\text{TP}\right)}{\left(\text{TP}+\text{FN}+\text{FP}\right)}X100. \end{array}$$

It is obvious from the table and graphical representation that the proposed method has a leading edge with respect to DSC value being 96.21%, whereas JSI value is 94.32% when compared to previous methods in brain tumor detection and classification. Dice similarity coefficient (DSC) values achieved by the proposed multilayered SVM with CNN are clearly superior to those achieved by earlier approaches.

Similarly, the parameters such as sensitivity, accuracy, specificity, and precision are provided in Table 2.

**Figure 16:**
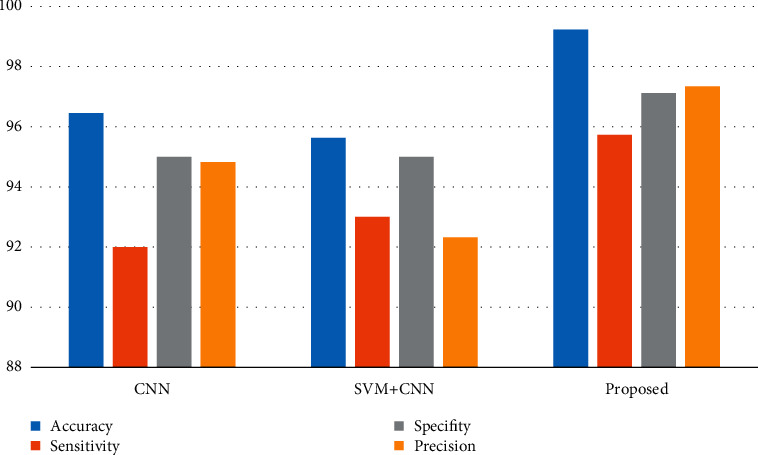
Graphical plot of sensitivity, accuracy, specificity, and precision.

The graphical representation for the comparison of the parameters such as sensitivity, accuracy, specificity, and precision is also provided in Figure 16.

In this case, the accuracy (ACC) parameter which is of significance is used to calculate the percentage value of the correct tumor region of interest classification rate, which is represented in equation (3), whereas sensitivity (SE) is used to estimate the exact percentage value of how sensitive the method is to compute the corresponding value of the tumor identification rate, and its equation is provided in equation (4).

(3) $$\begin{array}{c} \text{Accuracy }\left(ACC\right)=\frac{\left(\text{TP}+\text{TN}\right)}{\left(TP+TN\right)+\left(FP+FN\right)}X100, \end{array}$$

(4) $$\begin{array}{c} \text{Sensitivity }\left(\text{SE}\right)=\frac{\left(\text{TP}\right)}{\left(\text{TP}+\text{FN}\right)}X100. \end{array}$$

Also, the parameters specificity (SP) and precision (PR) are evaluated, where specificity (SP) discusses the rate value obtained between true negative (TN) values and true positive (TP) values represented as per the formulated equation (5) and further precision (PR) designates about the number of digits in terms of percentage that are used to present a value, and its equation to compute is shown in equation (6).

(5) $$\begin{array}{c} \text{Specificity }\left(\text{SP}\right)=\frac{\left(\text{TN}\right)}{\left(\text{TN}+\text{FP}\right)}X100, \end{array}$$

(6) $$\begin{array}{c} \text{Precision }\left(\text{PR}\right)=\frac{\left(\text{TP}\right)}{\left(\text{TP}+\text{FP}\right)}X100. \end{array}$$

It is obvious from the table and graphical representation that the proposed method has a leading edge with respect to accuracy by 99.23%, sensitivity by 95.73%, specificity by 97.12%, and precision by 97.34% when compared to previous methods in brain tumor detection and classification.

## 5. Conclusion and Summary

Various schemes for detecting and classifying brain tumors have been proposed and investigated in the literature in order to broaden therapy options and patient endurance. Brain tumor segmentation and detection have been processed in steps such as preprocessing, training, testing, and classification in this research work. The dataset's input images are first filtered and normalized for intensity values. Patch extraction, along with feature extraction, has been viewed as an intermediary stage in this work. After the characteristics were extracted, they were trained and tested in a CNN environment before being given to a multiple layer SVM classifier to display the tumor state in the MR scans. The fundamental advantage of CNN over its predecessors is that it automatically discovers significant features without the need for human intervention. For prior and proposed approaches, critical metrics such as dice similarity coefficient (DSC), Jaccard similarity index (JSI), sensitivity (SE), accuracy (ACC), specificity (SP), and precision (PR) are computed and compared. The proposed strategy proved to be an ideal solution with an accuracy of 99.23 percent. The methods such as gray-level co-occurrence matrix can also be employed in future along with the proposed methodology other than splitting to resolve the issues of losing any small details. The performance and evaluation of the proposed CNN involving multilayered SVM can be improved in the future by undertaking additional research and researching various deep networks. As a future scope, other deep networks can also be investigated for better classification rather than patches losing the details.

## Figures and Tables

**Algorithm 1:**
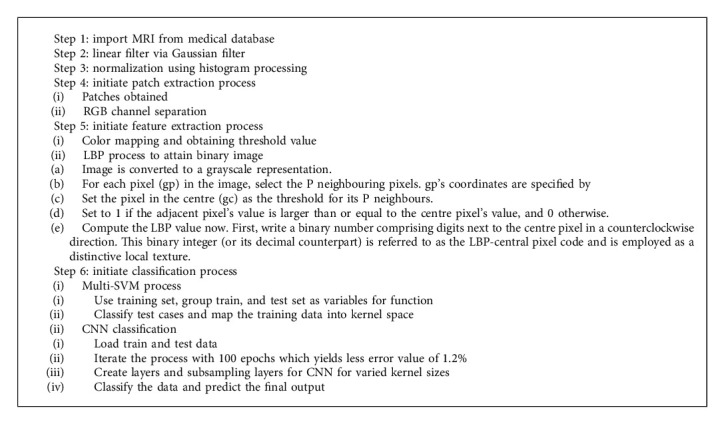


**Table 1:** Parametric comparison of DSC and JSI.

Classification methods	Dice similarity coefficient (DSC)	Jaccard similarity index (JSI)
CNN	83.85	89.2
SVM + CNN	88	90.41
Proposed multi-SVM + CNN	96.21	94.32

**Table 2:** Parametric evaluation and comparison.

Classification methods	Accuracy (%)	Sensitivity (%)	Specificity (%)	Precision (%)
CNN	96.45	92	95	94.82
SVM + CNN	95.63	93	95	92.32
Proposed multi-SVM + CNN	99.23	95.73	97.12	97.34

## Data Availability

The processed data are available upon request from the corresponding author.
